# Assessment of Knowledge, Attitude, and Practice Towards Migraine Among the General Population in the Kingdom of Bahrain

**DOI:** 10.7759/cureus.40928

**Published:** 2023-06-25

**Authors:** Zainab Ali, Hani Humaidan

**Affiliations:** 1 General Practice, Royal College of Surgeons in Ireland, Medical University of Bahrain, Busaiteen, BHR; 2 Neurology, Clinical Neurosciences, Salmaniya Medical Complex, Manama, BHR

**Keywords:** arabian gulf, headache disorders, neurological disease, bahrain, chronic migraine (cm)

## Abstract

Objective: The primary objective of this research is to assess the general population’s knowledge, attitude, and practice towards migraine.

Method: A cross-sectional study was conducted on a total sample of 255 participants. The data were obtained from a self-administered electronic questionnaire (Appendix) distributed through social media. Data analysis was done using the IBM Corp. Released 2019. IBM SPSS Statistics for Windows, Version 26.0. Armonk, NY: IBM Corp. For statistics, frequency, percentage, median, and standard deviation were calculated.

Results: Among the Bahraini population, knowledge about migraine definition, triggers, risk factors, symptoms, and treatment is satisfactory. However, knowledge about migraine prophylaxis is limited. Attitude and practice towards the disease showed adequate responses among the population, as they prefer consulting a doctor and believe that lifestyle modification is the best migraine treatment.

Conclusion: The general population of the Kingdom of Bahrain needs more awareness regarding migraine. Although the majority of the population showed good responses, which displayed some knowledge of the disease, community-based campaigns are still needed.

## Introduction

Migraine is the most common multidisciplinary and multifactorial neurological disorder characterized by recurrent headaches [1]. Both genetics and environmental risk factors play a significant role in the development of migraine [1]. These factors need to be observed to help reduce migraine incidence. Young individuals (25-34 years) reported the highest incidence rates at 23/1000 person-years in women and 10/1000 person-years in men, according to a Danish study [2].

Using international criteria, such as the International Classification for Headache Disorder Third Edition (ICHD-III), to diagnose migraine can lower the probability of misdiagnosis. ICHD was created by the International Headache Society in 2013 and is used in this study as a reference. Migraine is divided into two major types: migraine without aura and migraine with aura. Migraine without aura is a recurrent headache disorder manifesting in attacks lasting four to seventy-two hours. Typical characteristics of the headache are unilateral location, pulsating quality, moderate or severe intensity, aggravation by routine physical activity, and association with nausea and/or photophobia and phonophobia. Migraine with aura is characterized by recurrent attacks lasting minutes, unilateral site, and fully reversible visual, sensory, or other central nervous system symptoms that develop gradually and are usually followed by headache and associated migraine symptoms. Some patients also experience a prodromal phase, occurring hours or days before the headache, and/or a postdromal phase following the headache [3].

Migraine is the second-leading cause of disability worldwide, following back pain [4]. However, it is considered the leading cause of disability worldwide in people younger than 50 years, specifically women [4]. Additionally, migraine is associated with other comorbidities like depression, anxiety, epilepsy, obesity, and chronic pain in areas like the neck and lower back [4-6]. Moreover, some chronic diseases, such as asthma, chronic obstructive pulmonary disease, bronchitis, diabetes, hypertension, and dyslipidemia, are related to migraine [4]. Generally, comorbidities are more frequently associated with chronic migraine than with episodic migraine [4].

Migraine treatment is divided into acute and preventive phases. Acute therapy aims to stop attacks once they have started, prevent disability, and lessen migraine pain and related symptoms. Preventive therapy helps reduce the frequency and severity of expected attacks in those with a significant headache burden [7]. A successful treatment strategy must take into account the patient’s needs and expectations, the impact of the headache on their lives, their symptoms and co-morbidities, as well as knowledge of prior treatments. Successful headache control requires educating patients about their condition and the medications they are taking [7].

This study aims to assess the Bahraini population’s understanding of migraine and their attitudes and practices towards the disease.

## Materials and methods

This is a cross-sectional study conducted in the Kingdom of Bahrain from March 2022 to May 2022. A convenient sample of 257 participants was taken; two participants refused to take part in the study. A final sample of 255 participants was enrolled in the study.

Adults aged ≥ 18 years who are either Bahrainis or residents of Bahrain were included in the study. Non-Arabic, Non-English readers, and adults older than 75 years were excluded.

This was a questionnaire-based (Appendix) study where a digital questionnaire was designed after reviewing multiple scientific engines like UpToDate, Science Direct, and ICHD-III to form a simple, relevant questionnaire suitable for all educational levels. The governmental hospital’s ethical committee validated the questionnaire. Arabic and English versions of the survey were distributed to Arabic and English readers through social media and to shopping mall visitors. It was sent through WhatsApp and Airdrop to people who agreed to participate in the study. The questionnaire was composed of four main sections: demographic data, knowledge, attitude, and practice toward migraine disease. Demographic characteristics included age, gender, marital status, level of education, occupation, and nationality. The knowledge included migraine definition, triggers, risk factors, distribution, aura, treatments, and prophylaxis. The behavioral section comprised various questions to assess the importance of migraine and how an individual approaches it. 

After receiving all questionnaires from the candidates, data were collected, entered, and analyzed using the IBM Corp. Released 2019. IBM SPSS Statistics for Windows, Version 26.0. Armonk, NY: IBM Corp. For statistics, frequency, percentage, and median were calculated. 

Before the commencement of the form process, a written consent agreement was offered to the participants with an explanation of the study, their role, and their rights. All participants signed the agreement before enrollment. Ethical approval for the study was provided by the research committee for the governmental hospitals in the Kingdom of Bahrain. The approval number is 10240122.

## Results

Of all the respondents, 255 agreed to take part in this study, while two refused. Most of the participants (66.3%) were 18-38 years of age, and 29% of the respondents were 39-59 years of age. The median age was 1.0 (SD 0.5). Around three-fourths of the participants were female (73.3%), while males constituted only 26.3%. Most of the subjects were Bahrainis (83.5%), and around half of them were married (56.1%). The level of education of the majority is high, as 82.4% have a bachelor’s degree, and 15.3% have at least passed secondary school. Half of the subjects were employed (47.8%), while others were students, retired, housewives, or unemployed (Table 1).

**Table 1:** Demographics of the 255 subjects

Sociodemographic Variable	n (%)	
Age (in years)	18–38	169 (66.3)
	39–59	74 (29)
	60–75	12 (4.7)
Gender	Male	67 (26.3)
	Female	188 (73.3)
Nationality	Bahraini	213 (83.5)
	Non-Bahraini	42 (16.5)
Marital status	Single	110 (43.1)
	Married	143 (56.1)
	Divorced/Widowed	2 (0.8)
Education level	None	1 (0.4)
	Primary	0 (0)
	Intermediate	5 (2)
	Secondary	39 (15.3)
	University	210 (82.4)
Occupation	Employed	112 (47.8)
	Unemployed	23 (9)
	Housewife	15 (5.9)
	Student	59 (23.1)
	Retired	36 (14.1)

Analysis of the knowledge section is illustrated in Table 2. Interestingly, around 60% of the respondents defined migraine as a chronic neurological disease and understood its treatment, symptoms, and triggers like stress, chocolate, and flickering lights. The same percentage identified family history as an important risk factor for the disease. On the other hand, around 80% (SD 0.4) of the respondents were aware that women are more susceptible to migraine, had an idea about the pain characteristic as a unilateral headache, and chose nausea and photophobia as auras. Noticeably, only 19.2% (SD 1.1) showed any understanding of B-blockers as a prophylactic medicine (Table 2).

**Table 2:** Knowledge among 255 subjects

Knowledge	Correct
Migraine is a chronic neurological disease.	152 (59.6%)
Women are more likely to have migraine.	202 (79.2%)
The most common migraine triggers are stress, flickering lights and chocolate.	148 (58%)
Family members who suffer from migraine are common among migraine sufferers.	153 (60%)
Every person who has a migraine has certain symptoms that begin hours or days before the headaches start.	145 (56.9%)
Some medicines help alleviate migraine once it starts.	168 (65.9%)
Pain on one side is the most identifiable symptom of this type of headache.	212 (83.1%)
Migraine sufferers have either nausea or photophobia or both as aura(s).	228 (89.4%)
B-blockers are prophylaxis against migraine.	49 (19.2%)

Four questions were asked in the questionnaire to understand the general population’s practice toward migraine. Results revealed that 51.4% of the participants had suffered from migraines, and 48.6% had not. Moreover, 91% believe medications and lifestyle modification are the most convenient treatments for migraine and 92.9% claim that they would recommend their relatives and friends who suffer from migraine to consult a doctor. Furthermore, 34.9% stated that they would suggest a certain type of medication for them, as per Table 3. 

**Table 3:** Population’s practice towards migraine

Practice	n (%)
Have you ever suffered from migraine?	
Yes	131 (51.4%)
No	124 (48.6%)
Medications and lifestyle modification are the best treatments for migraine.	
Yes	232 (91%)
No	23 (9%)
If your friend or relative is suffering from a migraine, would you recommend that they consult a doctor?	
Yes	237 (92.9%)
No	18 (7.1%)
Will you suggest your friend a certain type of drug if he has a migraine?	
Yes	89 (34.9%)
No	166 (65.1%)

Further, attitudes towards migraine were measured for this population. In cases of recurrent headaches, 72.5% of the participants stated that they would consult a doctor, whereas the remaining quarter would opt to take paracetamol without consulting a doctor, and 2% would go to the pharmacist for help. Lifestyle modification was the treatment choice for 86.3% of participants. Additionally, more than half of the participants preferred having follow-up sessions for treatment, but 21.6% would not want to do so, as illustrated in Table 4. 

**Table 4:** Population’s attitude towards migraine

Attitude	n (%)
If you are suffering from recurrent headaches, what would you do?	
Consult a doctor	185 (72.5%)
Go to a pharmacist for help	5 (2%)
Take paracetamol by yourself without consulting a doctor	65 (25.5%)
If you suffer from migraine, will you modify your lifestyle?	
Yes	220 (86.3%)
No	35 (13.7%)
Will you attend follow-up sessions with a doctor for several months for migraine treatment?	
Yes	200 (78.4%)
No	55 (21.6%)

## Discussion

This is the first study in the Kingdom of Bahrain to assess citizens’ knowledge, attitudes, and practices towards migraine. Bahrain has a high population density [8]; therefore, evaluating knowledge among the Bahraini population should impact the quality of health services. The findings of this study will also serve as a turning point for subsequent studies assessing any required modifications in the community’s perception of migraine. 

Generally, knowledge of the disease among the Bahraini community was sufficiently acceptable, according to the results obtained. The results were similar to a previous study conducted in the Kingdom of Saudi Arabia (KSA) [9]. In our study, more than half of the participants knew that migraine affects women more than men. Furthermore, 83.1% identified unilateral headache as a main symptom of migraine, and the majority of participants knew about migraine treatment. In Saudi Arabia [9], 87.3% of their respondents correlate unilateral headaches with migraine, while half of them consider migraine to equally affect both men and women. Additionally, around 51.2% do not know about migraine treatment. Both studies show similar results considering the sample sizes, which are 255 and 385 in our study and their study, respectively. Another recent study in Saudi Arabia [10] showed similar results to ours with regard to public knowledge of migraine prophylaxis. While 14.7% of participants in KSA recognized Inderal (B-Blockers) as a preventive medicine for migraine, 17.9% stated the same in our study. The similarity of the results between the two previous studies in Saudi Arabia and the current one in Bahrain can be attributed to the similarity in customs and habits between the two nations. 

Internationally, a study in Germany [11] assessed people’s awareness of this chronic disease among their population. Results show good self-awareness among the migraineurs themselves. About 70% of the participants with migraine recognized their headache as a migraine, and 62.5% of them contacted a physician in the last 12 months for a diagnosis. 

In the same study [11], the level of education showed a correlation with knowledge; people with a high educational level displayed high self-awareness about migraine. In our study, 82.4% had a university education, which reflects public knowledge of migraine. However, in general, many people do not know if they have migraines, and this low self-awareness can affect their daily activities. 

Surprisingly, looking at migraine awareness among medical care providers reveals shocking results. A study in India showed that the level of awareness of the disease is very poor among medical students at Saveetha University [12]. Another study in Pakistan [13] revealed that although doctors were able to distinguish between the three main forms of headaches (Migraine, Tension, and Cluster), many migraine headaches were misdiagnosed as tension headaches. Additionally, limited knowledge about triptans was one of the factors that contributed to the minimal use of this therapeutic agent by doctors enrolled in the study. Moreover, a Turkish study showed that only 10.5% of primary physicians knew all the diagnostic standards for migraine [14]. A large cohort study conducted in a multi-headache center illustrates poor knowledge among patients and physicians in seven countries [15].

It is crucial to evaluate comprehension among those who provide information, diagnosis, and therapy to gauge understanding of a disease within a community. Physicians have a responsibility to raise public knowledge of all diseases, particularly considering the growing use of online search databases as a source of information.

Attitude and practice for this neurological disease are very good in Bahrain compared to nearby countries like Saudi Arabia [16], where the population attitude was negative as the respondents preferred not to consult a doctor, change their lifestyle, or even have a follow-up appointment with a doctor. In the current study, most participants prefer to do the opposite.

More than half of the participants in Saveetha [12] would not recommend consulting a doctor for their friends or relatives regarding migraine. However, 63.3% will suggest a drug to their friends to relieve migraines. This can be due to their medical educational background, as they choose the first option, which leads to the advice of a certain medication. On the other hand, the present study illustrates a high percentage of respondents who recommend doctor consultations and a low percentage who suggest a medicine. 

Chronic disease treatment is very difficult, especially in cultures with a high prevalence of self-medication. This is due to a lack of awareness. In the current study, 25.5% of participants preferred self-medication to ask for help from a doctor or pharmacist. However, the percentage of people who believe in self-medication for headache treatment is even greater in Saudi Arabia, where it reaches up to 41.3% [15]. 

All the aforementioned studies confirmed a lack of understanding among people regarding migraine. Conducted in various parts of the world, they have all come to the same conclusion: raising public awareness about migraine is crucial, as is also demonstrated in other studies in Sharjah and Italy [17,18]. 

This study has several limitations. The sample size was small in relation to the whole population, which might not faithfully reflect the Bahraini community. Recruiting a larger number of participants and extending the period of data collection to at least six months might solve this problem. Another limitation is the possibility of answering the questionnaire with the assistance of other people; hence, an accurate evaluation of knowledge level is difficult. Filling out the questionnaire via face-to-face interview should yield more accurate results. Despite the limitations, this is the first study in Bahrain assessing citizens’ knowledge, attitudes, and practices with regard to migraine. 

Our recommendation is to put more effort into improving public awareness about migraine. It is necessary to conduct more educational campaigns across all communities, focusing on the right attitudes and practices toward migraine. At the same time, doctors should be more educated about the disease, which helps in providing accurate information to patients, especially regarding treatments that help in understanding its chronic nature. 

## Conclusions

In sum, the level of knowledge, except on prophylaxis, is satisfactory in the Bahraini population. Furthermore, average attitudes and practices were adequate, considering the sample size. However, people still need more education on migraine, as it is a chronic disease that affects patients’ quality of life.

## Appendices

Appendix

Table 5 contains the research questionnaire.

**Table 5:** Research questionnaire

Q1. Gender								
Male				Female				
Q2. Age								
18-38	39-59		60-75				>75	
Q3. Education								
None	Primary		Intermediate			Secondary		University
Q4. Occupation								
Employed	Housewife		Student			Unemployed		Retired
Q5. Marital status								
Single	Married				Widowed/Divorced			
Q6. Nationality								
Bahraini		Non- Bahraini						
Q7. Migraine is								
Chronic Neurological Disease	Acute Neurological Disease		Acute Eyes Disease			None of the above		
Q8. Who is most likely to have migraines?								
Women	Men		Teens			Children		
Q9. What is a common trigger of migraines?								
Stress	Flickering lights		Chocolate and red wine			All of the above		
Q10. More than half of migraine sufferers have something in common. What is it?								
Allergies that affect their breathing	Excess weight		High blood pressure			Family members who have migraines		
Q11. Every person who has migraines has certain symptoms hours or days before the headache starts.								
True			False					
Q12. No medicines can help once a migraine has started.								
True			False					
Q13. Although migraines vary, what's one of the most identifiable symptoms of this kind of headache?								
Pupils dilate	Nausea		Pain on one side of the head			Dizziness		
Q14. What's one type of aura that migraine sufferers have?								
Body temperature rises	Body temperature falls		Sensation of a flashing light			Severe nausea		
Q15. Which of these is a medicine to help prevent migraines?								
Medicines to prevent seizures	Antidepressants		Beta-blockers			None of the above		All of the above
Q16. IF you suffer from recurrent headaches, what do you do?								
Consult a doctor	Go to pharmacist for help		Taking paracetamol by yourselves without consulting a doctor					
Q17. IF you suffer from migraine, will you modify your lifestyle?								
Yes			NO					
Q18. Will you go for follow up session to a doctor for several months for migraine treatment?								
Yes			NO					
Q19. Have ever suffer from Migraine?								
Yes			NO					
Q20. Medications and lifestyle modification is the best treatable way for migraine.								
YES			NO					
Q21. If if your friend or relative is suffering from migraine, would you recommend them consulting a doctor?								
YES			NO					
Q22. Will you suggest your friend certain type of drugs if he has migraine?								
YES			NO					
